# Contactless Cardiac Health Monitoring with Millimeter-Wave Radar Based on PMG-SATNet

**DOI:** 10.3390/s26092579

**Published:** 2026-04-22

**Authors:** Tianjiao Guo, Jianqi Wang, Nianzeng Yuan, Hao Lv, Fulai Liang, Zhiyuan Zhang, Jingzhe Wang, Yunuo Long, Huijun Xue

**Affiliations:** 1 School of Biomedical Engineering, Fourth Military Medical University, Xi’an 710032, China; tianjiaoguo@fmmu.edu.cn (T.G.); wangjq@fmmu.edu.cn (J.W.); nianzengy@fmmu.edu.cn (N.Y.); fmmulvhao@fmmu.edu.cn (H.L.); liangfulai@fmmu.edu.cn (F.L.); 2 School of Basic Medical Sciences, Fourth Military Medical University, Xi’an 710032, China; zhiyuanzhang@fmmu.edu.cn (Z.Z.); wangjingzhe@fmmu.edu.cn (J.W.); yunuolong@fmmu.edu.cn (Y.L.)

**Keywords:** cardiovascular diseases, contactless cardiac health monitoring, deep learning, ECG, millimeter-wave radar

## Abstract

Cardiovascular diseases are the primary causes of mortality worldwide, often characterized by subtle onset and acute progression. Traditional ECG electrodes may cause skin irritation, limiting routine monitoring and early risk assessment. Relying on the advantages of non-contact monitoring, millimeter-wave radar-based cardiac monitoring combined with deep learning has become a popular research direction recently. To overcome the poor generalization of methods trained from single-source datasets, this study designed seven experimental scenarios covering wakefulness and sleep. A novel deep learning network consisting of encoder and decoder structures named PMG-SATNet was proposed. The encoder comprises a parallel multi-scale feature extraction module and a global temporal relationship modeling module to capture fine-grained local patterns and long-range dependencies. The decoder employs a temporal convolutional network augmented with a spectral attention mechanism to emphasize clinically relevant ECG frequency bands and suppress respiration and body motion interference. After being validated on the self-built dataset, PMG-SATNet outperformed baseline models in terms of Pearson correlation coefficient and root mean square error, with an improvement of 3.3% and 3.8%, and 16.4% and 23.8%, respectively. The validation results imply that PMG-SATNet is capable of recovering ECG signals from millimeter-wave radar-derived chest vibrations with high fidelity and can potentially be implemented in real-life cardiac health monitoring.

## 1. Introduction

Cardiovascular diseases (CVDs) are one of the primary causes of disability and mortality worldwide, and have become a critical public health issue that impairs human life expectancy and hinders sustainable economic and social development. According to the Global Burden of Disease (GBD) series, the number of deaths from CVDs was estimated to reach 20.5 million by 2025, accounting for 26.8% of total deaths from all diseases. Estimates further suggest that this figure will surge to 35.6 million by 2050, marking a 73.4% increase and imposing considerable pressure on global healthcare systems [1,2]. However, the progression of CVDs often exhibits insidious and paroxysmal characteristics; without timely early warning and therapeutic intervention, irreversible severe consequences may occur. Therefore, the implementation of dynamic cardiovascular health monitoring, CVD risk assessment, and early diagnosis outside hospital settings are of significant importance in reducing mortality rates associated with CVDs [3].

An electrocardiogram (ECG) records human cardiac electrical activity and provides critical diagnostic information for CVDs, and is widely regarded as the clinical “gold standard” for assessing cardiovascular health [4]. Current ECG monitoring methods primarily rely on surface electrodes, which are attached to the chest, arms, or legs to capture the subtle electrical changes associated with the repolarization process following myocardial depolarization in each cardiac cycle. These electrical changes are used to record some characteristic waveforms, such as the P-wave representing atrial depolarization, the QRS complex representing ventricular depolarization, and the T-wave representing ventricular repolarization [5]. By analyzing ECG waveform morphology, physicians can effectively identify various abnormalities such as arrhythmia and myocardial ischemia, and subsequently design targeted interventions to prevent and control the progression of CVDs [6,7,8,9,10,11,12,13].

Although the electrode-based method for ECG monitoring is widely used in clinical practice, its reliance on skin-contact electrodes limits its applicability for routine out-of-hospital cardiovascular health monitoring and early risk assessment. Firstly, this electrode-based approach may induce skin irritation due to adhesive electrodes and restrict the wearer’s daily activities [14]. Secondly, certain cardiac conditions such as intermittent disorders like paroxysmal atrial fibrillation and asymptomatic myocardial ischemia require prolonged monitoring to capture their episodic dysfunction [15], yet the postural changes and increased skin moisture in wearers may lead to electrode detachment. Thirdly, multi-lead ECG devices necessitate frequent electrode replacement, which progressively increases the cost of long-term cardiac health monitoring and generates substantial medical waste [16]. Even Holter monitors, which support continuous recording for 24–48 h, still require multiple lead connections, leading to user inconvenience and potential electrode detachment. Moreover, Holter monitoring has poor resistance to motion artifacts, data analysis is typically performed offline, and it lacks real-time alert functionality [8,17,18]. In recent years, some lightweight wearable devices such as smart bands and smart watches have emerged and improved wearing convenience [17,19,20,21], but owing to limitations in signal quality and algorithmic accuracy, most of them can only provide coarse-grained heart rate estimation and fail to extract fine-grained electrophysiological features such as P-wave and T-wave, making it difficult to meet the requirements for CVD risk assessment and diagnosis [22]. Consequently, given the growing burden of CVDs and the diversity of out-of-hospital application scenarios, developing an ECG monitoring technology that integrates long-term continuity, wear comfort, high accuracy, and low cost has become a pressing and widespread healthcare need. 

Over the past decade, millimeter-wave radar has been extensively explored for applications including human vital signs detection, animal cardiopulmonary monitoring, and indoor health monitoring [23,24,25,26,27,28,29]. Compared to non-contact detection methods such as audio and video, millimeter-wave radar employs electromagnetic waves as the detection medium, enabling non-contact acquisition of vital signs such as respiration and cardiac activity. It can not only penetrate clothing, bedding, and similar materials, but is also unaffected by environmental noise or lighting conditions, thus providing a more advantageous solution for out-of-hospital non-contact cardiac health monitoring. Furthermore, given the advantages of millimeter-wave radar systems, including their compact size, low cost, and privacy protection, the radar-based non-contact cardiovascular health monitoring systems would show considerable practicality once commercialized [30,31,32]. Early radar-based cardiovascular health monitoring primarily focused on coarse-grained cardiac feature extraction, such as the detection of human heart rate and respiratory rate under static or slight bodily motion conditions, and the monitoring of heartbeat signals while suppressing respiratory harmonics and environmental noise interference. Commonly employed signal processing algorithms include Fourier Transform (FT), Wavelet Transform (WT), Empirical Mode Decomposition (EMD), Variational Mode Decomposition (VMD), and Multiple Signal Classification (MUSIC), among others [33,34,35]. Wang et al. utilized the VMD algorithm with millimeter-wave radar to extract the heartbeat signal, and estimated the exact timing of heartbeats by identifying its wave location within the extracted signals [36]. Jin et al. proposed a vital sign separation method based on improved Ensemble Empirical Mode Decomposition (EEMD) and Continuous Wavelet Transform (CWT), which effectively enhanced the signal-to-noise ratio (SNR) of radar echo signals, allowing for the accurate extraction of both respiratory and heartbeat signals [37]. The above methods can separate the primary respiratory and cardiac components to some extent, but their effectiveness in suppressing respiratory harmonics close to the heartbeat frequency remains inadequate, which consequently limits the accuracy of heartbeat monitoring. In addition, the extracted heartbeat signals exhibit significant waveform difference compared to contact-based ECG and thus have limited utility in advancing cardiac health monitoring.

Cardiac activity can be interpreted from two perspectives, namely cardiac electrical conduction and mechanical motion. Cardiac electrical conduction refers to the process in which electrical impulses are initiated at the sinoatrial node, propagate through the conduction system to cardiomyocytes, and trigger sequential repolarization and depolarization of the atria and ventricles. When recorded from the body surface, this rhythmic electrical activity manifests as the ECG waveform [5]. Cardiac mechanical motion refers to the physical process of coordinated contraction and relaxation of the atria and ventricles driven by the underlying electrical activity. It has been proven that cardiac mechanical activity can cause a slight vibration displacement in the body surface, and the millimeter-wave radar can extract the heartbeat signal by capturing the phase change in the echo signal triggered by the slight vibration. The aforementioned critical process that converts the electrical activity of cardiomyocytes into mechanical contractions is termed a cardiac excitation–contraction coupling (ECC) mechanism. In recent years, numerous studies based on the ECC mechanism and its related modeling have verified the existence of a highly synchronized yet nonlinear coupling relationship between cardiac mechanical motion and cardiac electrical activity from a physiological standpoint [38,39]. Early studies attempted to extract electrophysiological features from radar-derived cardiac mechanical motion signals using traditional signal processing methods such as FT and wavelet decomposition [40,41]. However, owing to these methods essentially relying on linear or stationarity assumptions, they struggle to fully capture the complex nonlinear mapping relationship between radar signals and ECG signals. Wang et al. adopted VMD combined with a lightweight deep neural network to achieve high-precision heart rate estimation using millimeter-wave radar signals. However, their method only extracts discrete cardiac pulses rather than reconstructing continuous ECG morphology, and thus cannot support clinical diagnostic needs based on waveform assessment such as P-wave, QRS complexes, or T-wave [42].

With the advancement of deep learning technology, a growing number of researchers have focused on modeling the nonlinear mapping relationship from millimeter-wave radar-based cardiac mechanical motion signals to ECG signals. Kumar et al. employed precise target localization (PTL) and a graph-attention-based HPR-Net to reconstruct high-quality heart pulse waveforms (HPW) from radar echoes of atrial fibrillation (AF) patients, diagnosing AF based on the arrhythmia reflected in the high-quality HPW. However, this study did not provide detailed information on the dynamic changes in the heart during the monitoring process [43]. Chen et al. first proposed a data-driven end-to-end framework for reconstructing ECG waveforms from millimeter-wave radar signals. This approach extracts 4D cardiac mechanical motion signals and employs a deep network to model their nonlinear relationship with electrical activity, achieving a Pearson correlation coefficient (PCC) of 0.86 compared with clinical ECG [44]. Building on this, a subsequent study introduced a cross-modal autoencoder that utilized a 1D-CNN and Conformer with a cardiac event predictor for nonlinear feature mapping, further improving the PCC to 0.914 [45]. More recently, a time-frequency fusion-based feature extraction module was proposed, along with joint time-domain and frequency-domain loss functions during training. This method not only achieved high accuracy heart rate estimation, with Mean Absolute Errors (MAE) as low as 0.20 bpm and 0.15 bpm, but also maintained stable reconstruction of ECG morphology [46]. Jin et al. proposed an attention-augmented deep convolutional generative adversarial network (Attention-DCGAN) with an improved K-means clustering method to reconstruct ECG signals from millimeter-wave radar echoes, achieving high-fidelity reconstruction with a PCC as high as 0.9853 [47]. These studies have achieved significant progress in radar-based ECG waveform reconstruction, addressing the purification of cardiac mechanical motion signals under the interference of respiratory harmonics with similar frequencies. However, most existing methods collect cardiac mechanical motion signals from the position directly in front of the chest under normal breathing conditions. These methods are trained on single-source datasets and thus exhibit poor generalization. They overlook the variation in human postures during the two primary states of wakefulness and sleep in real-world settings, as well as the characteristic differences in the corresponding ECG signals under varying postures and physiological states. As a result, it remains a challenge for these approaches to maintain adaptability in out-of-hospital continuous cardiac monitoring, such as in-home environments.

Inspired by the aforementioned research, a network named Parallel Multi-scale-Global Feature Learning with Spectral Attention-based Temporal Convolutional Network (PMG-SATNet) for radar–ECG reconstruction was proposed in this study. It achieved high accuracy modeling with the ability to reconstruct both temporal and spectral details of ECG signals. The research process comprised typical physiological data acquisition, data processing, and performance evaluation. The contributions of this work can be summarized as follows:

(1) Unlike the data acquisition scheme for cardiac mechanical and electrical signals in existing research, seven experimental scenarios that represent the primary static lifestyles of individuals in in-home environments, such as sitting (awake) and lying (asleep), were designed. In those scenarios, radar antennas were placed at different orientations relative to the human body to capture cardiac mechanical motion. The relatively rich dataset provides real data support for research on cardiac health monitoring methods in in-home environments.
(2) Based on the collected data under various postures and physiological states, a novel deep learning architecture named PMG-SATNet for radar-to-ECG mapping was designed in this study. Unlike existing feature extraction models that rely solely on either convolution or attention mechanisms, PMG-SATNet innovatively integrates multi-scale feature extraction and a spectral attention mechanism, and balances local feature modeling with the capture of long-term temporal dependencies. It significantly enhances the modeling capability of ECC across multiple states.
(3) Comprehensive performance evaluation and ablation studies were implemented. On the one hand, the results demonstrate that the proposed PMG-SATNet achieves superior capabilities in morphology, wave timing localization error, and intuitive reconstruction results under various postures and physiological states. On the other hand, the ablation studies further verify the effectiveness of each core module in the proposed model.

To present this work clearly, the rest of the paper is structured as follows: Section 2 introduces the hardware experimental platform, the design of data-acquisition scenarios, radar signal processing methods, and the framework of PMG-SATNet. Section 3 and Section 4 describe the self-built dataset in this study, present the obtained results, and conduct baseline comparisons and performance evaluations. Finally, this study concludes with a summary of the research and proposes future directions for improvement.

## 2. Materials and Methods

### 2.1. Hardware Experimental Platform

**Figure 1:**
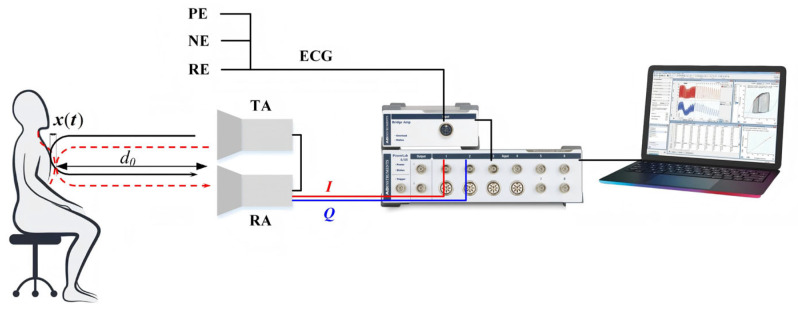
Schematic diagram of the experiment.

A 94 GHz millimeter-wave radar system (manufactured by ELVA-1 Company, Saint Petersburg, Russia), ECG electrode sensors, and a PowerLab multi-physiological signal acquisition module (ADInstruments International Trading Co., Ltd, Dunedin, New Zealand) are jointly utilized to construct a hardware experimental platform for synchronous acquisition of cardiac mechanical motion and cardiac electrical signal data. The main parameters of the radar are shown in Table 1. The schematic diagram illustrating the composition of the experimental platform and the principle of radar detection of human cardiac mechanical motion is shown in Figure 1. As illustrated in the figure, the radar transmits an electromagnetic wave via the transmitting antenna (TA) toward the subject’s chest. The reflected echo signal is captured by the receiving antenna (RA). The initial distance between the radar and the subject’s chest is denoted as $d_{0}$. When the electromagnetic wave reaches the chest, it undergoes a range delay and phase modulation due to chest wall vibration x(t) caused by cardiac mechanical activity. This vibration signal carries the micro-motion information of the heart. Meanwhile, the positive electrode (PE), negative electrode (NE), and reference electrode (RE) of the ECG are attached to the inner side of the subject’s left and right arms and above the right ankle, respectively, to collect the ECG signals. The echo signals from the in-phase (I) and quadrature (Q) channels of the radar are connected to analog channels 1 and 2 of the PowerLab module. Here, the I channel captures the signal component in-phase with the transmitted wave, while the Q channel captures the component with a 90-degree phase shift. Combining them avoids the detection blind spots inherent in using a single channel, thereby ensuring the complete extraction of cardiac micro-motion information. The ECG signal is amplified by a bio-amplifier, bandpass filtered at 0.5–40 Hz, and then connected to the third analog channel of the PowerLab module. This configuration ensures strict synchronization between the radar echo signal and the ECG ground truth signals, and avoids minor data bias due to manual signal alignment. After the two types of signals are collected, the signals from the PowerLab module are transmitted to a computer via a USB interface and displayed and stored synchronously using the matching LabChart 7.2. The LabChart interface allows manual control over the acquisition and storage of multi-channel physiological data. Before the experiment, the data sampling rate is set to 100 Hz.

### 2.2. Experimental Setup

To align with real-life scenarios for cardiac health monitoring under stable bodily conditions, a data acquisition procedure including various body postures and physiological states was designed, aiming to evaluate the performance of the proposed method for in-home monitoring. A total of 30 healthy subjects were recruited, with an age range from 19 to 45 years, and a balanced male-to-female ratio (1:1). All subjects were confirmed to be in good health based on medical history review, with no systemic, immunological, or dermatological diseases, thus meeting the inclusion criteria. Prior to participation, all subjects were fully informed of the study content and potential risks, and voluntarily provided written informed consent. Each subject performed the following seven scenarios:

- Sitting-CR-Eu: Upright sitting posture (Sitting), the chest facing the radar antenna (CR), under eupneic breathing (Eu).
- Lat Decub-BR-Eu: Lateral decubitus position (Lat Decub), the back facing the radar antenna (BR), under eupneic breathing (Eu).
- Lat Decub-CR-Eu: Lateral decubitus position (Lat Decub), the chest facing the radar antenna (CR), under eupneic breathing (Eu).
- Supine-CSR-Eu: Supine position (Supine), the lateral side of the chest facing the radar antenna to ensure detection of chest micro-motion induced by cardiac mechanical activity (CSR), under eupneic breathing (Eu).
- Supine-CSR-Ap: Supine position (Supine), the lateral side of the chest facing the radar antenna to ensure detection of chest micro-motion induced by cardiac mechanical activity (CSR), under intermittent apnea (Ap).
- Supine-CSR-Hy: Supine position (Supine), the lateral side of the chest facing the radar antenna to ensure detection of chest micro-motion induced by cardiac mechanical activity (CSR), under hypoxic breathing (Hy).
- Supine-CSR-RSB: Supine position (Supine), the lateral side of the chest facing the radar antenna to ensure detection of chest micro-motion induced by cardiac mechanical activity (CSR), under rapid shallow breathing (RSB).

Each subject repeated the above seven scenarios. The total cardiac data acquisition under different conditions lasted approximately 80 min per subject. A rest period of several minutes was provided between consecutive scenarios to allow physiological parameters to return to baseline before proceeding to the next one.

### 2.3. Methods and Models

#### 2.3.1. Signal Model of Radar

The continuous-wave radar detects cardiac mechanical motion based on the Doppler principle. In this study, the unmodulated signal emitted by the transmitting antenna is:

(1) $$T(t)=A\cos(2\pi f_{0}t+\varphi (t)),$$

where A is the signal amplitude, $f_{0}$ is the carrier frequency, and φ(t) is the phase noise. Assuming the initial distance between the radar and the target’s chest is $d_{0}$, when the radar electromagnetic wave reaches the target’s chest, it undergoes a range delay and phase modulation due to chest vibration x(t). The total propagation distance of the transmitted signal at this point is $2d(t)=2d_{0}+2x(t)$, and the corresponding time delay is $t_{d}=2d(t)/c$, where c is the speed of light. The phase modulation caused by the fixed distance $d_{0}$ is denoted as $\theta_{0}$, where λ is the wavelength. Therefore, the received signal can be expressed as:

(2) $$R(t)=KT(t-\frac{2d(t)}{c})=KA\cos\left[2\pi f_{0}(t-\frac{2d(t)}{c})+\varphi (t-\frac{2d(t)}{c})+\theta_{0}\right],$$

where K is the amplitude attenuation factor. The received signal is then mixed with the local oscillator signal and passed through a low-pass filter to obtain the baseband signal as:

(3) $$B(t)=\frac{KA^{2}}{2}\cos(\frac{4\pi d_{0}}{\lambda_{0}}+\frac{4\pi x(t)}{\lambda_{0}}+∆\varphi (t)-\theta_{0})=\frac{KA^{2}}{2}\cos\left[\theta +\frac{4\pi x(t)}{\lambda_{0}}+∆\varphi (t)\right],$$

where the constant phase term is $\theta =\frac{4\pi d_{0}}{\lambda_{0}}-\theta_{0}$, and $∆\varphi (t)=\varphi (t)-\varphi (t-\frac{2d_{0}}{c})$ represents the residual phase noise.

To avoid the issue of detection nulls in single-channel radar, a quadrature demodulation receiver is employed, utilizing both the in-phase (I) and quadrature (Q) outputs to ensure that at least one output channel is not located at a detection null. The output signal can be expressed as:

(4) $$I(t)=A_{I}\cos\left[\frac{4\pi x(t)}{\lambda_{0}}+\theta (t)\right],$$

(5) $$Q(t)=A_{Q}\sin\left[\frac{4\pi x(t)}{\lambda_{0}}+\theta (t)\right].$$

Here, $\theta (t)=∆\varphi (t)+\frac{4\pi \Delta d}{\lambda_{0}}$, when $A_{I}=A_{Q}$, the phase w(t) can be extracted by:

(6) $$w(t)=\arctan\frac{Q(t)}{I(t)}=\frac{4\pi x(t)}{\lambda_{\varrho }}+\theta (t).$$

The displacement x(t) and amplitude A(t) of the chest vibration signal can be expressed as:

(7) $$x(t)=\frac{\lambda_{0}}{4\pi }(w(t)-\theta (t)),$$

(8) $$A(t)=\sqrt{I^{2}(t)+Q^{2}(t)}.$$

#### 2.3.2. Signal Preprocessing

1. Radar Signal Preprocessing

The raw radar echo signals contain both respiratory and cardiac mechanical motion components. As respiratory harmonics partially overlap with the cardiac frequency band, a Maximal Overlap Discrete Wavelet Transform (MODWT) based on the db4 wavelet was employed to suppress respiratory interference [48]. The mixed signal was decomposed into multiple levels. The layers primarily associated with cardiac motion and those related to respiratory motion were automatically identified based on their spectral energy waves. In cases where automatic identification failed due to individual variability or low signal-to-noise ratio (SNR), the system automatically selects the key layers whose frequency spectrum falls within a predefined frequency band to reconstruct the cardiac mechanical motion signal, thereby preserving the primary cardiac components.

Subsequently, to further enhance the SNR and waveform consistency of the separated cardiac motion signal, the Adaptive Kalman Filter (AKF) was introduced [49]. This method integrates a state-space model with a real-time noise estimation mechanism. Both the process noise covariance and the measurement noise covariance were reasonably initialized, and a forgetting factor close to 1 was set to balance the weights between old and new data. Furthermore, cardiac frequency information was incorporated with a certain weighting to guide the filtering process. During recursive filtering, the system dynamically adjusted the noise covariance in real time based on signal characteristics, thereby effectively tracking time-varying noise.

2. ECG Signal Preprocessing

ECG signals are susceptible to various noises during acquisition, such as baseline drift, electromyographic (EMG) interference, and powerline interference. These noises can obscure key physiological features like the P-wave, QRS complex, and T-wave that may affect subsequent feature extraction and reconstruction accuracy. So, baseline wander removal and electrode noise suppression algorithms were employed sequentially to suppress noise while maximally preserving the physiological structure and dynamic characteristics of the original ECG signal. The ECG preprocessing steps are as follows.

- Baseline Drift Removal

To effectively eliminate baseline drift induced by respiratory movement and postural changes, the least-squares method was utilized to fit a fifth-order polynomial function that characterizes the baseline trend [50]. This fitted baseline trend was subtracted from the original signal—a stationary ECG signal free of baseline drift was obtained.

- Electrode Noise Suppression

To further suppress noise originating from poor electrode-skin contact, a wavelet thresholding method based on the statistical properties of noise was adopted. During signal processing, the noise standard deviation was estimated using the median absolute deviation (MAD) of the signal, and an adaptive soft threshold was then computed by integrating this estimate with the signal length. Wavelet coefficients with absolute values below the threshold were set to zero, while those that exceeded the threshold were subjected to soft threshold shrinkage [51].

#### 2.3.3. ECG Reconstruction Model

**Figure 2:**
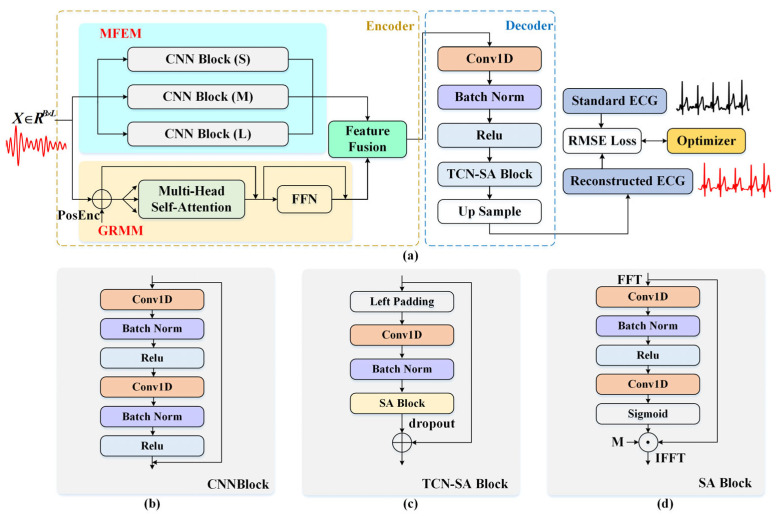
Architecture of the PMG-SATNet and detailed diagrams of its key modules. (**a**) Overall architecture of the PMG-SATNet; (**b**) the CNNBlock; (**c**) the TCN-SA Block; (**d**) the SA Block.

To achieve high accuracy end-to-end reconstruction of ECG signals from cardiac mechanical motion signals acquired by radar, a novel deep learning network named PMG-SATNet was proposed. The model employs an encoder–decoder architecture, as illustrated in Figure 2. The encoder comprises three core components, where the Multi-scale Feature Extraction Module (MFEM) and Global Relation Modeling Module (GRMM) are designed to capture fine-grained local patterns and long-range dependencies within the cardiac mechanical signal, and a Feature Fusion unit achieves efficient feature integration. The decoder consists of a TCN-SA block, where a Temporal Convolutional Network (TCN) is incorporated with Spectral Attention (SA), which decodes the fused features and suppresses noise to reconstruct standard ECG waveform with physiologically accurate P-wave, QRS complex, and T-wave morphologies.

1. Multi-scale Feature Extraction Module

The MFEM comprises three parallel branches, whose primary function is to extract multi-scale local features from the preprocessed cardiac mechanical motion signal. For each input segment with a duration of 10 s $X\in R^{B\times L}$, where B denotes the batch size and L = 1000 represents the signal sampling length, this module is structured around three distinct feature extraction branches with selected scales. The selection is based on the periodic characteristics of the input signal to capture short-duration pulsatile details, periodic rhythmic fluctuations, and long-term trend components, respectively.

In the first branch, each CNNBlock (S) employs a convolutional layer with a kernel, padding, dilation and stride, which are set to 3, 1, 1, and 1 respectively. The stacking of multiple such blocks yields a total receptive field spanning approximately 0.1 s. This constrained temporal scale enables the capture of fine-grained waveform details, such as those in the P-wave and T-wave, while avoiding the feature-smoothing effect of larger receptive fields.

In the second branch, each CNNBlock (M) employs a convolutional layer with a kernel, padding, dilation and stride, which are set to 7, 3, 1, and 1 respectively. Stacking these blocks yields a receptive field of approximately 0.25 s, which is designed to encompass the typical duration of a QRS complex, thereby enabling precise extraction of its key morphological features.

In the third branch, each CNNBlock (L) employs a convolutional layer with a kernel, padding, dilation and stride, which are set to 7, 6, 2, and 1 respectively. After stacking multiple blocks, the total receptive field corresponds to an actual duration of approximately 0.5 s. This larger receptive field scale facilitates the capture of longer-term rhythmic features, such as R-R intervals and HRV.

The outputs of the three parallel scale-specific branches are concatenated along the channel dimension and passed through a two-stage convolutional fusion layer, which compresses the channel dimension and applies a nonlinear transformation. Finally, 1D max pooling is applied for downsampling to fix the temporal length to *l*. The final output of this module is $F_{M}\in R^{B\times l}$.

2. Global Relation Modeling Module

To capture long-term dependencies and rhythmic evolution patterns in input segmentatiom, a Transformer-based GRMM is introduced as a global temporal feature extractor. This module is primarily composed of Positional Encoding (PosEnc), Multi-Head Self-Attention (MHSA), and a Feed-Forward Network (FFN).

The input $X\in R^{B\times L}$ is combined with a learnable PosEnc to retain temporal order information:

(9) $$X_{P}=X+P.$$

The encoded features $X_{P}$ are passed through a dropout layer to mitigate overfitting, followed by *m* stacked global relation layers. Each layer consists of MHSA and FFN. The MHSA is used to establish dynamic dependencies between arbitrary time steps. The computation for the *i*-th attention head is formulated as:

(10) $$H_{i}=Softmax\left(\frac{Q_{i}K_{i}^{T}}{\sqrt{d_{head}}}\right)V_{i}.$$

The outputs of all heads are concatenated, linearly projected, and then combined with a residual connection and layer normalization (LN):

(11) $$X_{M}=LN(X_{P}+Dropout(HW_{O})),$$

where $H=Concat(H_{1},\ldots ,H_{h})$ represents the concatenated results from all attention heads, and $W_{O}$ denotes the projection weight matrix. The FFN is employed to further model the nonlinear relationships among features and generate dynamic gating signals:

(12) $$G=\sigma (W_{g_{2}}\;ReLU(W_{g_{1}}X_{M})),$$

(13) $$X_{G}=LN(X_{M}+Dropout(G⊙X_{M})).$$

Here, $W_{g_{1}}$ and $W_{g_{2}}$ denote the weight matrices of the FFN, and σ represents the sigmoid function. The gating mechanism adaptively reweights features. Both MHSA and FFN incorporate residual connections and LN to stabilize training. After *m* layers, the output of this module is $F_{G}\in R^{B\times l}$ with an enhanced temporal modeling capability. Compared to the standard Transformer, the GRMM employs a more computationally efficient and lightweight design.

3. Feature Fusion Unit

The two extracted features are first projected into a shared embedding space via 1D convolutional layers: $F_{M}^{\prime }=\mathrm{ReLU}(W_{M\;}F_{M})$; $F_{G}^{\prime }=\mathrm{ReLU}(W_{G}F_{G})$. These projections are then concatenated along channel-wise to form $F_{\mathrm{CAT}}$, which is subsequently processed by a convolutional block for deep fusion:

(14) $$F_{\mathrm{FS}}=W_{F}\;\mathrm{ReLU}(\mathrm{BN}(W_{C}F_{\mathrm{CAT}})).$$

Here, $W_{C}$ is used to compress the channel dimension, while $W_{F}$ is applied to produce the final fused feature. Crucially, instead of performing naive addition or concatenation, it learns how to optimally integrate multi-scale local details within a global temporal context.

4. TCN-SA

After encoding the essential ECG characteristic, a decoder is required to map the fused feature $F_{\mathrm{FS}}$ back into the ECG signal and simultaneously filter out residual interference such as respiratory and motion artifacts. The decoder is constructed from a convolutional layer and multiple stacked TCN-SA blocks.

Assume the input after the convolutional layer as ${F^{\prime }}_{\mathrm{FS}}={\left[x_{0},\;x_{1},\;\ldots ,\;x_{n-1}\right]}^{T}$, where *n* is the sequence length, and $x_{t}\;\;(t\;=\;0,\;1,\;\ldots ,\;n-1)$ denotes the feature at time step t.

- TCN

The TCN combines causal convolutions with residual connections to model temporal dependencies and prevent gradient vanishing. The output at time step t is:

(15) $$y_{t}=BN(\overset{k-1}{\underset{i=0}{\sum }}w_{i}\cdot x_{t-i\cdot d}),$$

where d is the dilation rate, k is the kernel size, and $w_{i}$ are convolutional weights. The output sequence $Y_{\mathrm{TCN}}\;=\;{\left[y_{0},\;y_{1},\ldots ,y_{n-1}\right]}^{T}$ is subsequently fed into the SA block.

- SA

The SA block performs noise suppression through frequency-domain filtering and channel weighting. Firstly, $Y_{\mathrm{TCN}}$ is transformed to the frequency-domain via FFT:

(16) $$S_{f}=FFT(Y_{\mathrm{TCN}}).$$

The amplitude spectrum and phase spectrum are then derived:

(17) $$|S_{f}|=abs(S_{f}),\phi =angle(S_{f}).$$

A frequency-domain mask M is defined to attenuate non-ECG bands:

(18) $$\;M(f)=\{\begin{array}{ll} 1 & \mathrm{if}\;f\;\in \;[0.5,\;40]\;\mathrm{Hz}\\ 0.1 & \mathrm{otherwise} \end{array}.$$

A channel-attention weight $W_{SA}\;$ is generated via a two-stage 1D convolution and sigmoid function:

(19) $$\;W_{SA}=\sigma (Conv1d(ReLU(BN(Conv1d(|S_{f}|))))).$$

The weighted amplitude spectrum is obtained as:

(20) $$\;|S_{f}{|}^{\prime }=|S_{f}|⊙M⊙W_{SA}.$$

Combined with the original phase spectrum, the modified frequency representation is transformed back to the time-domain via an inverse Fourier transform (IFFT):

(21) $$\;\hat{S}=IFFT(|S_{f}{|}^{\prime }\cdot e^{j\phi }).$$

A residual connection is then applied by adding the filtered and enhanced feature with the original feature, ensuring feature integrity and preventing the loss of information due to filtering:

(22) $$\;Y_{\mathrm{SA}}=\hat{S}+Y_{\mathrm{TCN}}.$$

After stacking k TCN-SA Blocks, the final output is integrated with ${F^{\prime }}_{\mathrm{FS}}$ through a residual connection and then upsampled to reconstruct the ECG signal.

5. Loss Function

To ensure the reconstructed ECG signal closely matches the ground truth signal in both morphology and fine details, the Root Mean Square Error (RMSE) loss is employed:

(23) $$L_{RMSE}=\sqrt{\frac{1}{L}\sum_{i=1}^{L}({\hat{y}}_{i}-y_{i}{)}^{2}},$$

where L is the signal length, $y_{i}$ is the ground truth value, and ${\hat{y}}_{i}$ is the corresponding reconstructed value at time step *i*.

6. Morphological Metrics

To quantitatively assess the morphological errors between the reconstructed ECG and the ground truth ECG, three metrics including RMSE, PCC and MAE are employed.

PCC measures the linear correlation between the reconstructed signal $\hat{y}$ and ground truth signal y, ranging from −1 to 1, with a value close to 1 indicating strong linear correlation and high morphological similarity. It is defined as:

(24) $$L_{PCC}=\frac{\sum_{i=1}^{L}({\hat{y}}_{i}-\bar{\hat{y}})(y_{i}-\bar{y})}{\sqrt{\sum_{i=1}^{L}({\hat{y}}_{i}-\bar{\hat{y}}{)}^{2}}\cdot \sqrt{\sum_{i=1}^{L}(y_{i}-\bar{y}{)}^{2}}},$$

where $\bar{\hat{y}}$ and $\bar{y}$ are the means of $\hat{y}$ and y respectively.

MAE quantifies the average absolute deviation between the reconstructed and ground truth signals, providing a direct measure of reconstruction error:

(25) $$L_{MAE}=\frac{1}{L}\sum_{i=1}^{L}\left|{\hat{y}}_{i}-y_{i}\right|.$$

A smaller MAE indicates a lower average absolute deviation, reflecting higher waveform morphological reconstruction accuracy and closer amplitude agreement.

## 3. Results

### 3.1. Dataset and Details

Following the experimental setup described in Section 2.2, cardiac data were collected under various postures and physiological states. Each data segment was trimmed to a length of 10 s. After excluding segments affected by electrode detachment and significant motion artifacts, approximately 7.52 million frames (equivalent to about 21 h) of radar-based cardiac motion data and their synchronized ECG were retained, comprising around 7521 valid segments. The proportion of data corresponding to each experimental condition is presented in Table 2.

The proposed PMG-SATNet in this study was developed based on the PyTorch 2.0.0 and trained on an NVIDIA RTX 4090 GPU (24 GB). The training process employed was the Adam optimizer, incorporating an early stopping mechanism [52] and a cosine annealing learning rate scheduler for dynamic learning rate adjustment [53]. The initial learning rate was set to 0.001, with a total of 300 training epochs and a batch size of 64. For the dataset involving 30 subjects, a five-fold cross-validation strategy was adopted, where each fold used 24 subjects for training and the other six subjects for testing. Critically, all data segments belonging to a given subject were exclusively allocated to either the training or test partition, with no overlap between sets. This subject-level partitioning strategy avoids the inverse crime scenario and ensures a rigorous evaluation. To evaluate the temporal wave localization accuracy of the reconstructed ECG signals, the peak positions of the Q, R, S, and T waves were detected using the open source ECG analysis toolkit NeuroKit2 [54]. The same toolkit was applied uniformly to both the ground truth ECG signals and the reconstructed ECG signals to ensure independent and objective annotation. For each wave type, the absolute temporal error was calculated as the absolute difference between the peak position detected in the reconstructed signal and that detected in the ground truth signal.

### 3.2. Results Analysis

Through comparison analysis and ablation studies, this study systematically validates the performance advantages of the proposed model and the necessity of each module from three dimensions: morphology, wave timing localization error, and intuitive reconstruction results. The RMSE, PCC, and MAE are adopted as morphological evaluation metrics. Wave localization error is assessed using the median and 90 percentile of the localization errors of four typical ECG waves (Q, R, S, T). In addition, the experimental results are presented more comprehensively through cumulative distribution function (CDF) plots, box plots, and visual reconstruction comparisons. The classic MMECG and RadarNet which exhibit relatively high reconstruction accuracy are selected as baseline models [44,46]. The MMECG shares a similar main architecture with the proposed model: it employed an encoder that combines 1D convolutions and Transformers for feature extraction, and a TCN for decoding. The RadarNet adopts a pure CNN architecture, with both its encoder and decoder constructed based on improved ResNet convolutional blocks.

#### 3.2.1. Comparison Analysis

1. Morphological Evaluation

Morphological metrics directly reflect the waveform reconstruction accuracy. Lower RMSE and MAE values, along with a PCC value closer to 1, indicate a higher agreement between the reconstructed signal and the ground truth signals. The three aforementioned ECG reconstruction models are trained and tested using the same data partitioning method with five-fold cross-validation. The average test results for RMSE, PCC, and MAE are presented in Table 3. As shown, PMG-SATNet achieves a RMSE reduction of 16.4% and 22.1% compared to the MMECG and RadarNets, respectively. Similarly, its MAE is reduced by 28.2% and 31.7% compared to these two models, while its PCC increases to 91.79%. These results demonstrate that the proposed PMG-SATNet exhibits high morphological consistency with the ground truth ECG signals.

**Figure 3:**
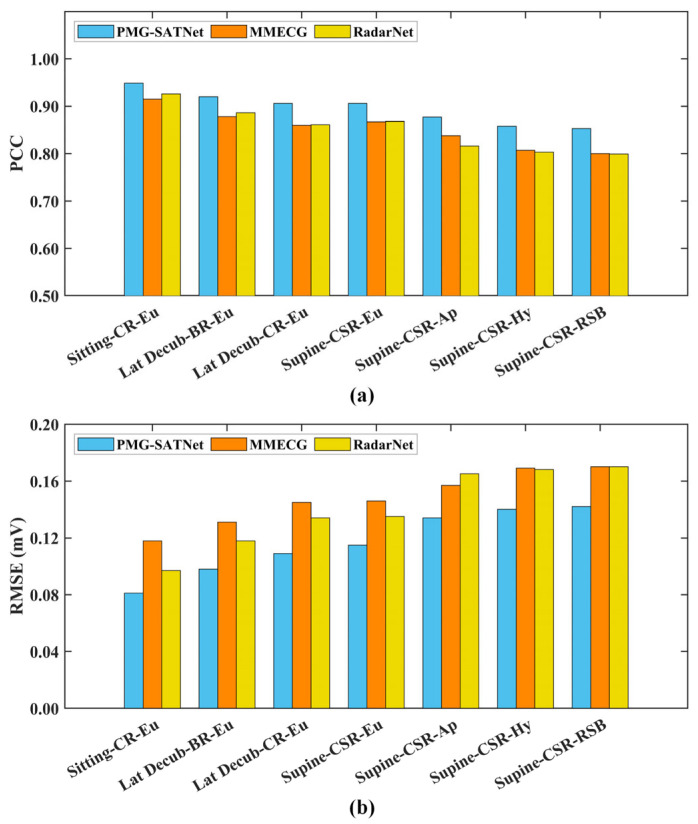
Comparison of PCC and RMSE obtained from the three models. (**a**) PCC comparison results; (**b**) RMSE comparison results.

To evaluate the robustness of the proposed model in diverse in-home heart health monitoring scenarios, a bar chart comparing the PCC and RMSE obtained from three models across seven physiological states is shown in Figure 3. As can be seen from Figure 3a, the PMG-SATNet achieves a PCC as high as 0.95 under the Sitting-CR-Eu state, and also maintains stable PCC values above 0.85 under the Supine-CSR-Ap, Supine-CSR-Hy, and Supine-CSR-RSB states. In contrast, the PCC results for these three states from the baseline models drop to around 0.80. As shown in Figure 3b, the RMSE of the PMG-SATNet is only 0.08 under the Sitting-CR-Eu state. While the RMSE increases under the Supine-CSR-Ap, Supine-CSR-Hy, and Supine-CSR-RSB states, ranging between 0.13 and 0.15, these values remain lower than those of the baseline models under the same three states, whose values are generally above 0.16.

**Figure 4:**
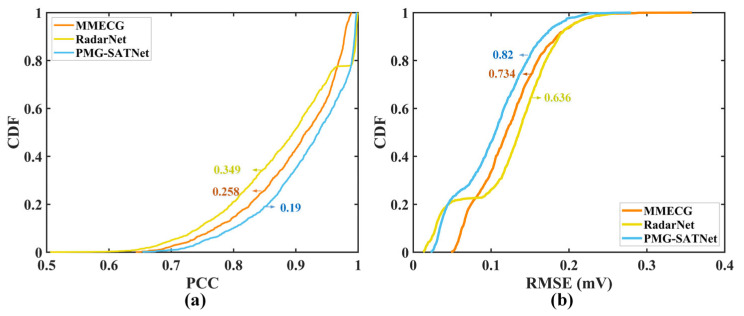
CDF plots for the three models: (**a**) cumulative distribution curves of PCC; (**b**) cumulative distribution curves of RMSE.

To further evaluate the overall performance stability of the proposed model, CDF analysis is performed on the PCC and RMSE. The CDF curves of PCC and RMSE are shown in Figure 4. As observed in Figure 4a, the cumulative probability for PMG-SATNet achieving PCC = 0.85 is 19%, outperforming comparisons to the MMECG and RadarNet of 25.8% and 34.9%. From Figure 4b, when RMSE = 0.15, the cumulative probability for PMG-SATNet reaches 82%, while the corresponding values for the other two baseline models are only 73.4% and 63.6%. These results indicate that the PMG-SATNet exhibits superior stability in morphological reconstruction across different samples.

2. Temporal Wave Localization Error

The accurate localization of Q-, R-, S-, and T-waves in ECG signals is crucial for clinical diagnosis. This study employs the median error and the 90 percentile error to evaluate the temporal localization accuracy of the reconstructed ECG signals. The comparison results of the temporal localization errors of the four waves between the reconstructed ECG signals from the three models and the ground truth signals are presented in Table 4. As can be observed, the PMG-SATNet achieves a median temporal localization error of 0.005 s and a 90 percentile error of 0.011 s for the R-wave, representing a reduction of 16.7–35.3% compared to the baseline models. Similarly, the PMG-SATNet achieves smaller median and 90 percentile errors of the Q-, S-, and T-waves.

**Figure 5:**
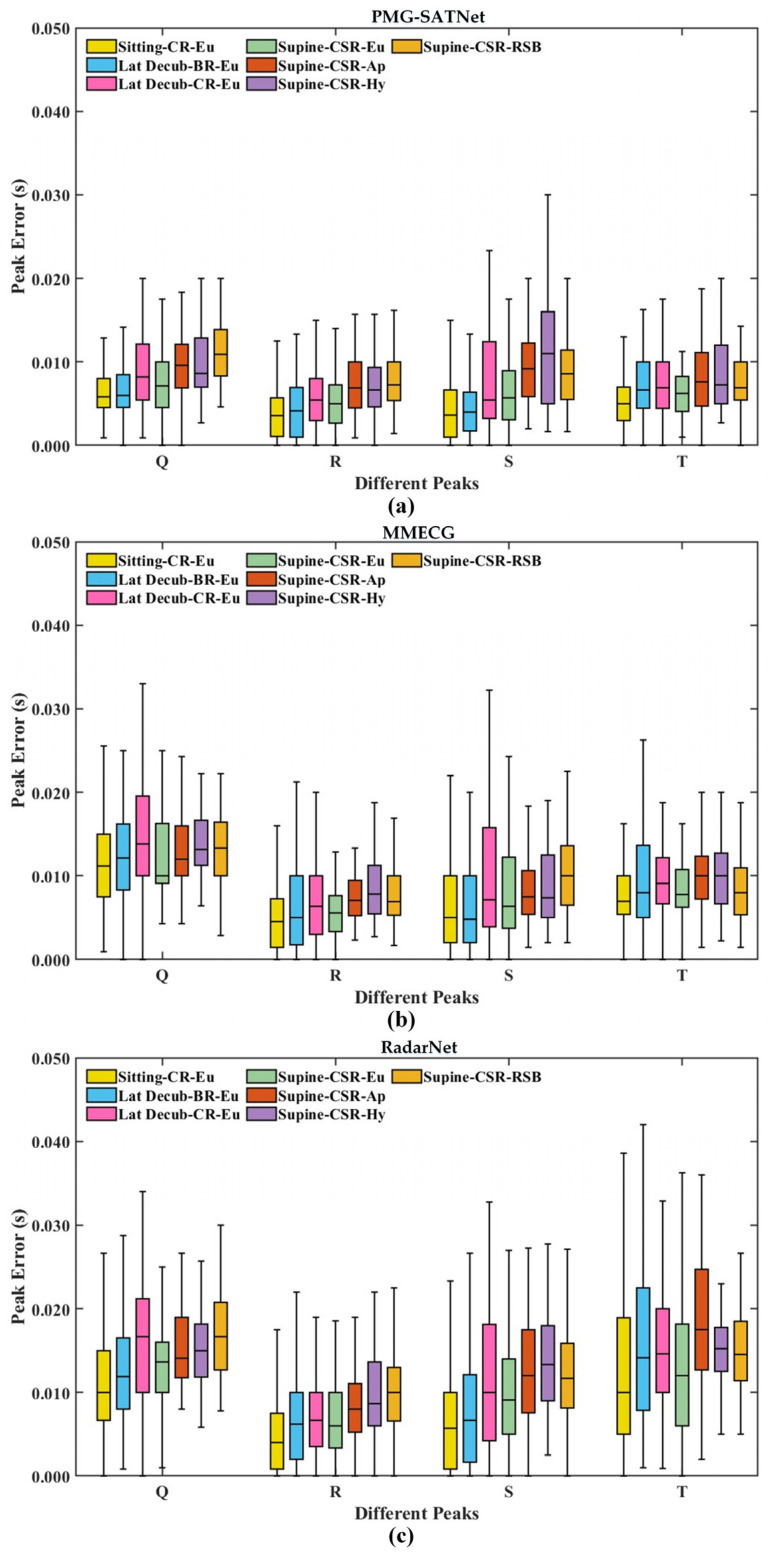
Box plots of wave localization errors of the three models under different states. (**a**) PMG-SATNet box plot; (**b**) MMECG box plot; (**c**) RadarNet box plot.

Box plots illustrating the temporal error across seven physiological states are shown in Figure 5. It can be observed that the wave localization errors of Q-, R-, S-, and T-waves for the PMG-SATNet model under all states consistently achieve lower errors with a relatively balanced error distribution. Regarding the R-wave, which is the most critical characteristic wave in ECG signals, the median localization error of the PMG-SATNet model remains within the range of 0.003–0.007 s, representing a reduction of up to 40.7% compared to the baseline models. Likewise, the localization errors of Q-, S-, and T-waves are lower.

3. Visual Reconstruction Results

**Figure 6:**
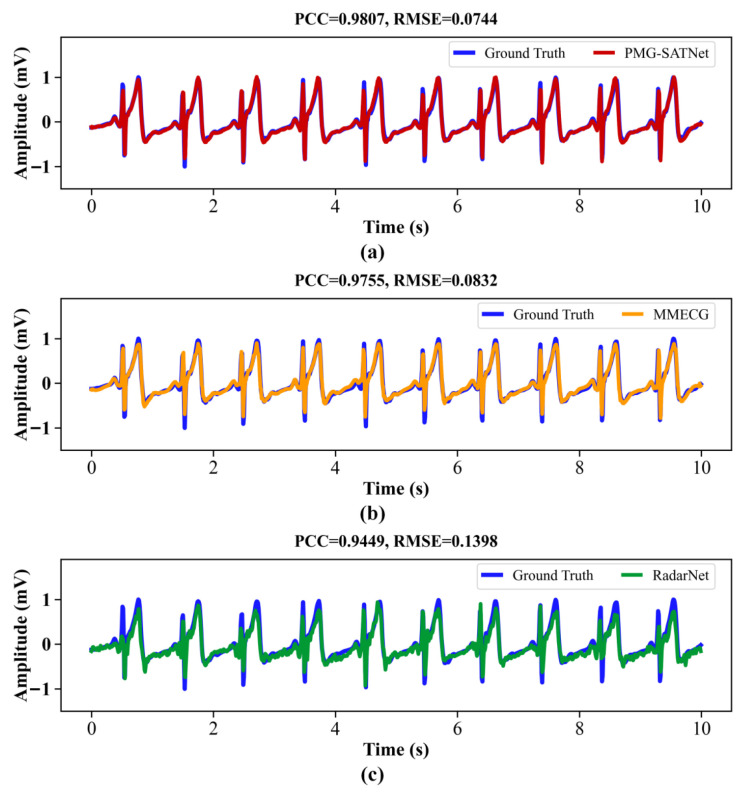
Comparison of reconstruction results. (**a**) PMG-SATNet reconstructed signal against the ground truth signal; (**b**) MMECG reconstructed signal against the ground truth signal; (**c**) RadarNet reconstructed signal against the ground truth signal.

Representative reconstructed ECG waveforms derived from the three models for the same sample are displayed in Figure 6. As can be observed, the ECG signal reconstructed by the PMG-SATNet (solid red line) almost completely coincides with the ground truth signals (solid blue line), demonstrating considerable consistency in morphological features such as waveform inflection points, the QRS complex, and T-wave details. The ECG signal reconstructed by the MMECG (solid orange line) exhibits a certain degree of waveform deviation in the QRS complex region. In contrast, the signal reconstructed by the RadarNet (solid green line) shows more pronounced waveform and amplitude deviations in both the QRS complex and T-wave regions, along with noticeable discrepancies in high-frequency fluctuations compared to the ground truth signals. These qualitative observations are further supported by quantitative metrics such as PCC and RMSE obtained from the three models, which also confirm the superior performance of the PMG-SATNet network in reconstructing fine-grained details.

#### 3.2.2. Ablation Studies

To validate the effectiveness of each core module in the proposed PMG-SATNet, four ablation models were designed: Model-1, which removes the GRMM from the original model; Model-2, which removes the MFEM; Model-3, which changes the connection between the MFEM and GRMMs from parallel to sequential; Model-4, which removes the SA module from the decoder of the original model. By comparing these ablation models with the original model, the contribution of each module to the designed architecture is quantitatively analyzed.

1. Morphological Evaluation

The average morphological evaluation results of the four ablation models are presented in Table 5. It can be observed that, under the condition of using the same decoder (TCN-SA), the PMG-SATNet model with the parallel combination of MFEM and GRMM in the encoder achieves a higher consistency between the reconstructed ECG signal and the ground truth signal. For models already equipped with the GRMM in the encoder, adding the parallel MFEM reduces the MAE value from 0.076 to 0.056. This indicates that incorporating the parallel MFEM further enhances the accuracy of ECG reconstruction by capturing subtle waveform characteristics of the ECG signal. Yet, comparing Model-4 and the complete PMG-SATNet model, although their encoders are identical, the spectral attention mechanism in the decoder plays a non-negligible role, improving the PCC value by approximately 1.33%.

**Figure 7:**
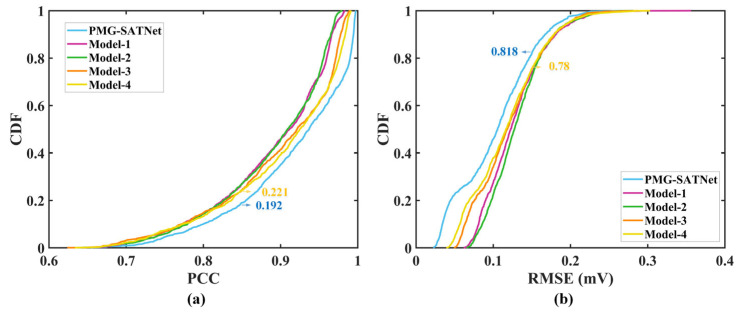
CDF plots for PMG-SATNet and the ablation models: (**a**) cumulative distribution curves of RMSE; (**b**) cumulative distribution curves of PCC.

The CDF curves of PCC and RMSE for the PMG-SATNet model and the four ablation models are shown in Figure 7. From Figure 7a, the CDF curve of PCC for the PMG-SATNet model consistently lies below those of the other four ablation models. When PCC = 0.85, the cumulative probability of PCC for the PMG-SATNet model shows an improvement of at least 13.1% compared to other ablation models. As seen in Figure 7b, the CDF curve of RMSE for the PMG-SATNet model consistently lies above those of the ablation models, particularly when RMSE = 0.15, and PMG SATNet model’s cumulative probability increases by least 4.87% compared to other ablation models.

2. Temporal Wave Localization Error

The above five models’ median and 90 percentile values of the temporal localization errors of the Q-, R-, S-, and T-waves between the reconstructed ECG signals and the ground truth signals are presented in Table 6. It can be observed that, under the condition of using the TCN-SA decoder, the PMG-SATNet model with the parallel combination of MFEM and GRMM in the encoder achieves the smallest temporal localization errors across all four waveform types. Model-1, which lacks the GRMM, exhibits larger wave localization errors compared to the other ablation models. Furthermore, although Model-4 and the PMG-SATNet model both have the parallel MFEM-GRMM combination in the encoder, owing to the spectral attention mechanism in the TCN decoder, the temporal localization errors of all four waveform types of the PMG-SATNet model are further reduced.

## 4. Discussion

The PMG-SATNet outperforms the baseline models MMECG and RadarNet in terms of morphological reconstruction accuracy, wave timing localization precision, and robustness across diverse scenarios. Its superior performance does not derive from stacking complex structures, but rather from the model’s effective extraction of ECG signal features and its alignment with the requirements of practical monitoring scenarios. Although the overall architecture design of the proposed model shares some similarities with MMECG, an additional MFEM is incorporated into the model encoder: this module constructs multiple parallel 1D convolutional branches with different receptive fields, and captures the morphological features of the ECG waveform at various temporal scales simultaneously. The addition of a multi-scale perception mechanism can effectively mitigate the local localization bias issue inherent in traditional single-branch structures with fixed receptive fields. RadarNet primarily relies on a singular 1D convolutional neural network architecture for reconstruction. Although 1D convolutions offer advantages in capturing local temporal patterns, their inherent limitation in local receptive fields makes it difficult to model long-term temporal dependencies within signals. Furthermore, ECG signals are intrinsically non-stationary time-series whose physiological detail lies not only in local waveform but also involves global temporal dynamics and rhythmic patterns. If the local segments of an ECG signal are solely focused on, it tends to lose global rhythmic information during the model training process. To address this problem, GRMM is incorporated into the PMG-SATNet model; this module performs temporal modeling on the input sequence using a lightweight Transformer structure, and learns long-term dependencies and dynamic evolution patterns along the time dimension.

In terms of the datasets for the three models, this study designed seven experimental scenarios involving various postures and physiological states to simulate real-world in-home radar-based cardiac health monitoring. The radar detects cardiac mechanical motion from multiple angles, rather than solely from a frontal chest position. Theoretically, the Supine-CSR-Ap state that simulates sleep apnea, the Supine-CSR-Hy state that simulates hypoxic breathing, and the Supine-CSR-RSB state that simulates rapid shallow breathing due to specific diseases, increases the difficulty of radar-based cardiac motion detection. Firstly, in these three states, the radar is placed laterally to the subject, detecting only chest vibrations caused by the heart on the lateral profile, resulting in less useful information compared to detection from the frontal chest position. Secondly, states involving breath-holding, hypoxia, and rapid shallow breathing represent varying degrees of systemic hypoxia. To compensate for oxygen deficiency, the sympathetic nervous system is activated, and heart rate is increased to enhance oxygen delivery efficiency. Obviously, the data corresponding to accelerated heart rate constitute a smaller proportion of the overall dataset as shown in Table 2, and the amplitude may exhibit subtle variations depending on the degree of hypoxia. This also leads to relatively lower reconstruction accuracy for ECG signals by all three models under the Supine-CSR-Ap, Supine-CSR-Hy, and Supine-CSR-RSB states, as detailed in Figure 3.

**Figure 8:**
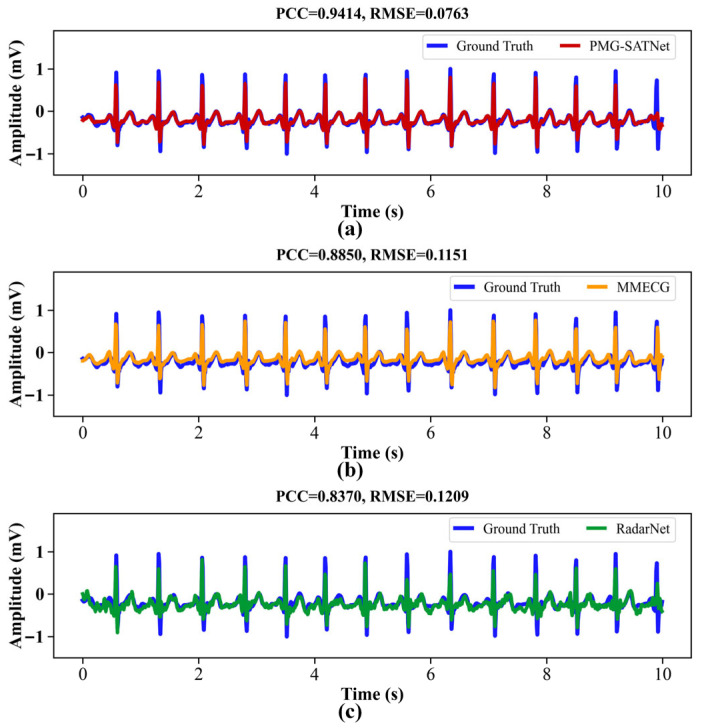
ECG reconstructed waveforms for the three models under the Supine-CSR-Ap state: (**a**) PMG-SATNet reconstructed signal against the ground truth signal; (**b**) MMECG reconstructed signal against the ground truth signal; (**c**) RadarNet reconstructed signal against the ground truth signal.

**Figure 9:**
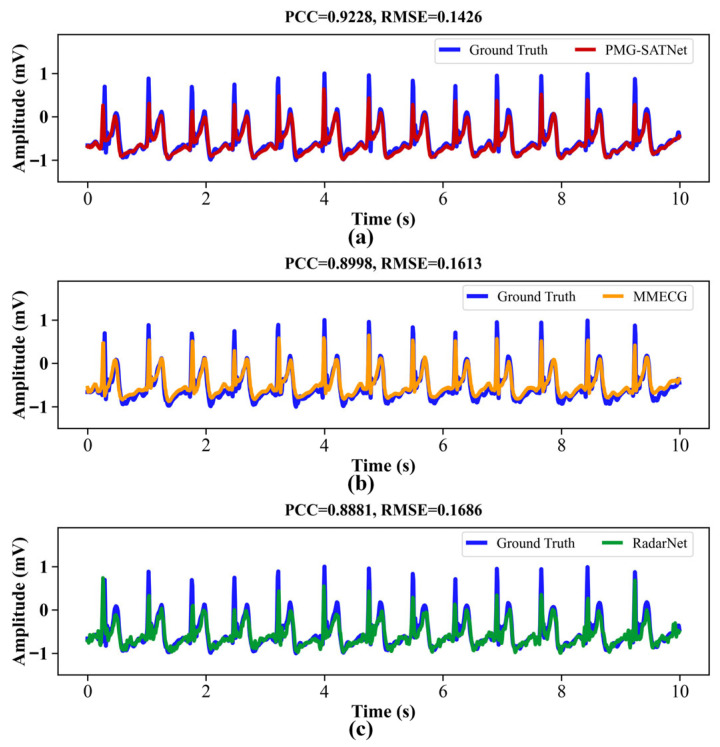
ECG reconstructed waveforms for the three models under the Supine-CSR-Hy state: (**a**) PMG-SATNet reconstructed signal against the ground truth signal; (**b**) MMECG reconstructed signal against the ground truth signal; (**c**) RadarNet reconstructed signal against the ground truth signal.

**Figure 10:**
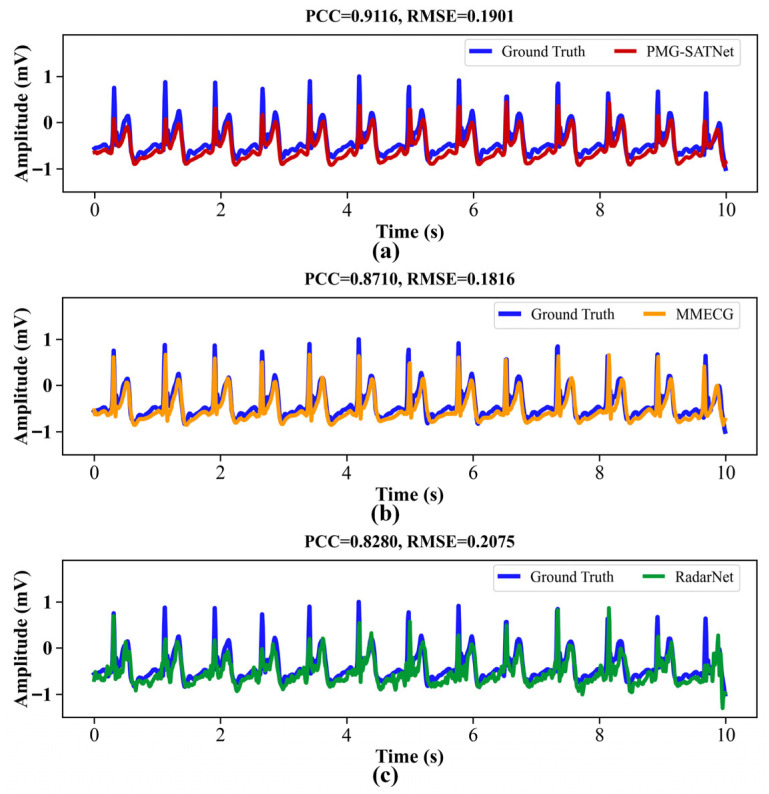
ECG reconstructed waveforms for the three models under the Supine-CSR-RSB state: (**a**) PMG-SATNet reconstructed signal against the ground truth signal; (**b**) MMECG reconstructed signal against the ground truth signal; (**c**) RadarNet reconstructed signal against the ground truth signal.

The ECG waveforms reconstructed by the three models under the Supine-CSR-Ap, Supine-CSR-Hy, and Supine-CSR-RSB states are displayed in Figure 8, Figure 9 and Figure 10. It can be observed that the heart rate in these states is faster than the resting state in Figure 5. Furthermore, in the Supine-CSR-Hy and Supine-CSR-RSB states, subjects unconsciously increase their breathing amplitude to enhance oxygen uptake, which in turn reduces the amplitude of chest vibrations caused by cardiac motion. Consequently, for these states, the amplitude reconstruction errors in the ECG signals increase for all three models. Additionally, the reconstructed signals from the MMECG and RadarNet exhibit imprecise detail reconstruction and increased interference components. To address these problems, the proposed model employs the MFEM for multi-scale waveform feature extraction combined with the GRMM for global temporal modeling, and introduces a spectral attention mechanism to suppress interfering frequency components. This approach partly compensates for the shortcomings of the baseline models in terms of scale adaptability and temporal completeness for ECG reconstruction tasks.

## 5. Conclusions

Millimeter-wave radar is not restricted by environmental noise and lighting conditions, enabling non-contact detection of cardiac mechanical motion in humans, demonstrating significant potential for future out-of-hospital heart health monitoring. Most existing studies on ECG reconstruction from cardiac mechanical motion based on millimeter-wave radar predominantly rely on limited and single-scenario datasets. Consequently, the resulting ECG reconstruction methods often lack adaptability to real-life scenarios. To further advance the application of radar technology in out-of-hospital cardiac health monitoring, this study designed a series of experimental scenarios involving various human postures and different breathing patterns, and collected a corresponding dataset. A novel deep learning network consisting of encoder and decoder structures named PMG-SATNet was proposed to achieve high accuracy conversion from cardiac mechanical motion signals to ECG signals. To comprehensively validate model performance, training and testing were conducted using a self-built dataset. Experimental results indicate that the proposed model outperforms existing baseline methods in terms of overall ECG morphological similarity and key wave localization accuracy. Ablation studies further verify the effectiveness of each core module in the proposed PMG-SATNet. A limitation of the current study is its exclusive focus on data acquired from healthy subjects. Pathological conditions, such as arrhythmias, atrial fibrillation, and myocardial infarction, present a more severe challenge, as the inherent regularization mechanisms of deep learning may fail in the presence of such abnormalities. Therefore, future research should prioritize expanding both the scale and diversity of the dataset, with a particular emphasis on integrating real-world clinical data from patients with cardiac abnormalities. This extension will be critical to further improving the model’s clinical practicality, robustness, and cross-population generalizability in real diagnostic scenarios.

## Figures and Tables

**Table 1:** Parameters of the 94 GHz radar system.

Transmit Frequency (GHz)	Transmit Power (mW)	Antenna Gain (dB)	Wavelength (mm)	Antenna Type
94	100	41.7	3	Cassegrain Antenna

**Table 2:** Proportion of cardiac data under various postures and physiological states.

Experimental Condition	Data Quantity	Proportion
Sitting-CR-Eu	2816	37.44%
Lat Decub-BR-Eu	1199	15.94%
Lat Decub-CR-Eu	1077	14.32%
Supine-CSR-Eu	968	12.87%
Supine-CSR-Ap	571	7.59%
Supine-CSR-Hy	458	6.09%
Supine-CSR-RSB	432	5.74%

**Table 3:** Performance comparison for ECG reconstruction.

Framework	Encoder	Decoder	PCC	RMSE	MAE
MMECG	CNN + Transformer	TCN	88.83%	0.122	0.078
RadarNet	ResNet	ResNet	88.38%	0.131	0.082
PMG-SATNet	MMEF + GRMM	TCN-SA	**91.79%**	**0.102**	**0.056**

**Table 4:** Comparison of Q-, R-, S-, and T-wave localization errors of the three models.

Framework	Percentile	Q	R	S	T
MMECG	Median	0.012	0.006	0.006	0.008
	90 percentile	0.020	0.014	0.018	0.020
RadarNet	Median	0.013	0.006	0.008	0.014
	90 percentile	0.022	0.017	0.021	0.029
PMG-SATNet	Median	**0.007**	**0.005**	**0.005**	**0.006**
	90 percentile	**0.014**	**0.011**	**0.016**	**0.015**

**Table 5:** Morphological evaluation comparison for ablation study.

Framework	Encoder	Decoder	PCC	RMSE	MAE
Model-1	MFEM	TCN-SA	89.40%	0.124	0.073
Model-2	GRMM	TCN-SA	89.56%	0.125	0.076
Model-3	MFEM - GRMM	TCN-SA	90.09%	0.119	0.071
Model-4	MFEM + GRMM	TCN	90.46%	0.115	0.068
PMG-SATNet	MFEM + GRMM	TCN-SA	**91.79%**	**0.102**	**0.056**

**Table 6:** Temporal wave localization error comparison for ablation study.

Framework	Percentile	Q	R	S	T
Model-1	Median	0.012	0.006	0.007	0.010
	90 percentile	0.020	0.014	0.020	0.043
Model-2	Median	0.010	0.005	0.006	0.008
	90 percentile	0.021	0.012	0.017	0.020
Model-3	Median	0.009	0.005	0.005	0.009
	90 percentile	0.017	0.013	0.020	0.025
Model-4	Median	0.011	0.005	0.006	0.007
	90 percentile	0.019	0.011	0.016	0.017
PMG-SATNet	Median	**0.007**	**0.005**	**0.005**	**0.006**
	90 percentile	**0.014**	**0.011**	**0.016**	**0.015**

## Data Availability

The data presented in this study are available on request from the corresponding author.
